# CpG oligodeoxynucleotide stimulates production of anti-neutrophil cytoplasmic antibodies in ANCA associated vasculitis

**DOI:** 10.1186/1471-2172-9-34

**Published:** 2008-07-14

**Authors:** Plinio R Hurtado, Lisa Jeffs, Jodie Nitschke, Mittal Patel, Ghafar Sarvestani, John Cassidy, Pravin Hissaria, David Gillis, Chen Au Peh

**Affiliations:** 1 Renal Unit, Royal Adelaide Hospital, North Terrace, Adelaide, 5000, Australia; 2 Institute of Medical and Veterinary Science, Frome Road, Adelaide, 5000, Australia; 3 The University of Adelaide, South Australia

## Abstract

**Background:**

Wegener's Granulomatosis and Microscopic Polyangiitis are life-threatening systemic necrotizing vasculitides of unknown aetiology. The appearance of circulating antibodies to neutrophil cytoplasmic antigens (ANCA) is strongly associated with the development of the disease. A link between infection and disease has long been suspected, and the appearance of ANCA antibodies has been reported following bacterial and viral infections. The depletion of circulating B cells with monoclonal antibody therapy can induce remission, and this observation suggests a pathogenic role for B cells in this disease. As bacterial DNA is known to induce B cell proliferation and antibody production via TLR-9 stimulation, we have explored the possibility that unmethylated CpG oligodeoxynucleotide, as found in bacterial and viral DNA, may play a role in stimulating circulating autoreactive B cells to produce ANCA in patients with vasculitis.

**Results:**

We have confirmed that unmethylated CpG oligonucleotide is a potent stimulator of antibody production by PBMC *in vitro*. The stimulation of PBMC with CpG oligonucleutides resulted in the production of similar amounts of IgG in both ANCA+ patients and normal controls. In spite of this, PR3 ANCA+ patients synthesised significantly higher amount of IgG ANCA than normal controls. In MPO ANCA+ patients, there was a tendency for patients to produce higher amount of ANCA than controls, however, the difference did not reach significance. Furthermore, we were able to detect circulating MPO-reactive B cells by ELISpot assay from the peripheral blood of 2 MPO+ ANCA vasculitis patients. Together, this indicates that circulating anti-neutrophil autoreactive B cells are present in ANCA+ vasculitis patients, and they are capable of producing antibodies in response to CpG stimulation. Of note, CpG also induced the production of the relevant autoantibodies in patients with other types of autoimmune diseases.

**Conclusion:**

Circulating ANCA autoreactive B cells are present in patients with ANCA^+ ^vasculitis. The production of ANCA from these cells in response to unmethylated CpG stimulation lead us to propose that stimulation of these cells by immunostimulatory DNA sequences such as CpG oligodeoxynucleotide during infection may provide a link between infection and ANCA associated vasculitis. This phenomenon may also apply to other antibody mediated autoimmune diseases.

## Background

The presence of circulating anti-neutrophil cytoplasmic antibodies (ANCA) has been found to be strongly associated with the development of Wegener's Granulomatosis and Microscopic Polyangiitis [1,2], commonly known as ANCA associated vasculitis. These conditions are characterised by systemic necrotising vasculitis, arthralgia, myalgia, inflammation of the upper and lower respiratory tracts, and acute necrotising glomerulonephritis. Proteinase-3 (PR3) and myeloperoxidase (MPO) are the most common neutrophil antigens targeted [3,4]. Notably, ANCA specificity in these patients is directed against either PR3 or MPO but seldom both. Infrequently, other neutrophil-derived antigens such as elastase and cathepsin G can instead be targeted.

Although the pathogenic role of ANCA remains controversial, there is increasing evidence that B cells and ANCA autoantibodies are important. For example, the depletion of CD20+ peripheral B cells with Rituximab can bring about remission in patients with refractory ANCA+ vasculitis [5]. In many though not all patients, the titre of serum ANCA mirrors disease activity [6,7]. Furthermore, plasma exchange may have a therapeutic role [8]. An animal model of ANCA associated vasculitis has been attempted by raising antibodies to MPO in MPO^-/- ^knockout mice [9]. These antibodies which had been raised against MPO as a neo-antigen caused necrotizing glomerulonephritis when injected into wild type mice.

The strongest evidence for ANCA in the pathogenesis of vasculitis resides in *in vitro *experiments [10,11]. They demonstrated binding of ANCA to cytokine-primed neutrophils resulting in neutrophil activation and degranulation, with the consequent release of reactive oxygen species and inflammatory cytokines, and subsequent cellular damage to neighbouring endothelial cells.

Another potential role of circulating IgG autoantibodies in the pathogenesis of antibody-mediated autoimmune disease may be to assist B cells and dendritic cells in the capture of autoantigens and subsequent presentation to T cells, thus contributing to the development and maintenance of autoantibody production [12,13].

In the case of ANCA associated vasculitis, infection has been suspected to play an important role in development of the disease. Friedrich Wegener first suggested the possible association of infection with Wegener's Granulomatosis while describing the disease [14,15]. Extensive studies have been carried out demonstrating the association of carriage of *staphylococcus aureus *in the nasal passages and Wegener's Granulomatosis [16]. This finding may be relevant to the manifestation of clinical pathology within the sinuses and the upper respiratory tract. In addition, the appearance of ANCA has been regularly reported in association with other infections such as bacterial endocarditis (anti-MPO by ELISA [17], and dual anti-MPO and PR3 by ELISA [18]), leprosy (determined by immunofluorescence only) [19], tuberculosis (mainly anti-PR3) [20], hepatitis C (anti-bactericidal/permeability increasing protein or Cathepsin G) [21], and parvovirus B19 (anti-MPO or PR3) [22].

Pendergraft and colleagues have reported that mice injected with antisense DNA-encoded PR3 generated antibodies that in turn stimulated anti-idiotypic antibodies, which recognised sense DNA-encoded PR3. Based on this phenomenon, they proposed that peptides derived from microbial organisms, which shared sequence homology with antisense DNA-encoded PR3, may serve as potential antigens to initiate autoimmunity [23].

In view of 1) the possible aetiological connection between infection and this disease, 2) the fact that Toll-like receptors (TLRs) play a critical role in the innate recognition of infectious pathogens by the immune system [24,25] 3) B cells are known to respond to viral and bacteria derived TLR-ligands such as CpG oligodeoxynucleotides, and 4) experimental evidence in murine animal models showing that TLRs are important to the development of several autoimmune disorders (reviewed in [26]), we wanted to examine the effect of TLR stimulation on the production of ANCA in vasculitis patients.

In this study, we focus on the effect of TLR stimulation, in particular the stimulation of PBMCs by the TLR-9 specific ligand single-stranded DNA with CpG motifs, CpG oligodeoxynucleotide (CpG) as found in bacteria and viruses, to see if this agent might stimulate *in vitro *production of IgG ANCA from patients with ANCA+ vasculitis. CpG sequences of bacterial and viral origin are hypomethylated in contrast to mammalian DNA which is predominantly methylated. It is thought that this difference in methylation status is one of the ways by which humans avoid responding to self-DNA [26]. We chose to concentrate on TLR-9 because TLR-9 is expressed mainly by B cells and plasmacytoid dendritic cells in humans [27] and, stimulation of TLR-9 by CpG is known to induce B cell proliferation and antibody production *in vitro *and *in vivo *[28].

## Results

### CpG-B induces PBMCs to produce ANCA in vasculitis patients *in vitro*

**Figure 1:**
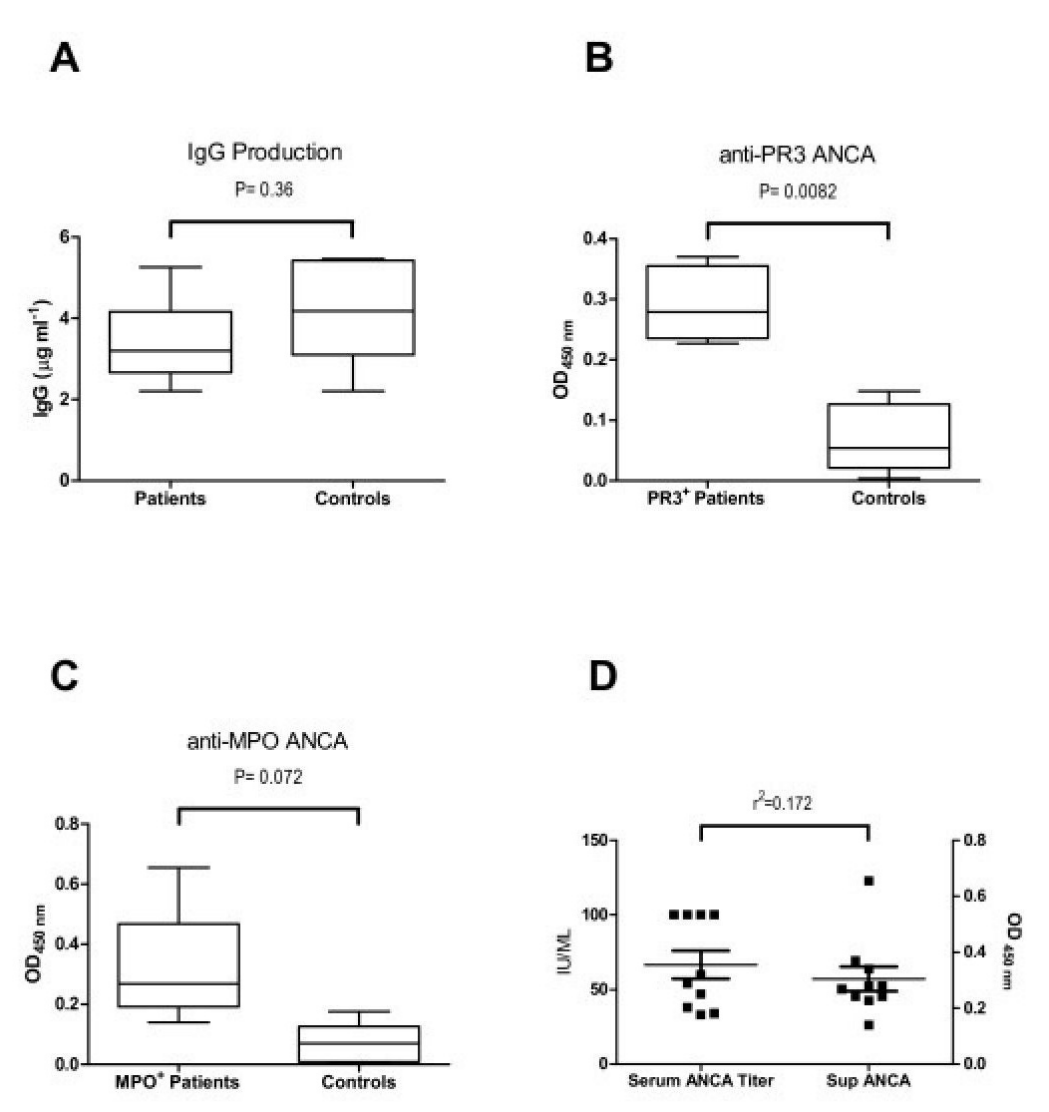
**CpG-B induces ANCA production from vasculitis patients *in vitro***. PBMCs isolated from patients with active ANCA associated vasculitis, 5 PR3^+ ^and 5 MPO^+ ^ANCA patients, were cultured with CpG-B and IL-2. Each patient assay was paired with a healthy control. After 12 days of culture, supernatants were harvested. IgG concentration and supernatant reactivity to either PR3 or MPO was measured by ELISA. The amount of IgG detected in the supernatants was of 5.5 ± 2.2 μg mL^-1 ^in the patients compared to 4.1 ± 1.2 μg mL^-1 ^in the control group (**A)**. Figure **B **shows the reactivity of the supernatants from PR3^+ ^ANCA patients towards PR3 antigen. The difference against control individuals was highly significant (P = 0.0082). Figure **C **shows the reactivity of the supernatants from MPO^+ ^ANCA patients towards MPO antigen. The difference was not significant (P = 0.072) although their supernatants showed a clear tendency towards higher reactivity compared to controls. There was no correlation between patients' serum ANCA titre at the time of the assay and their *in vitro *production of ANCA in response to CpG-B as shown in **D **(r^2 ^= 0.172).

**Figure 2:**
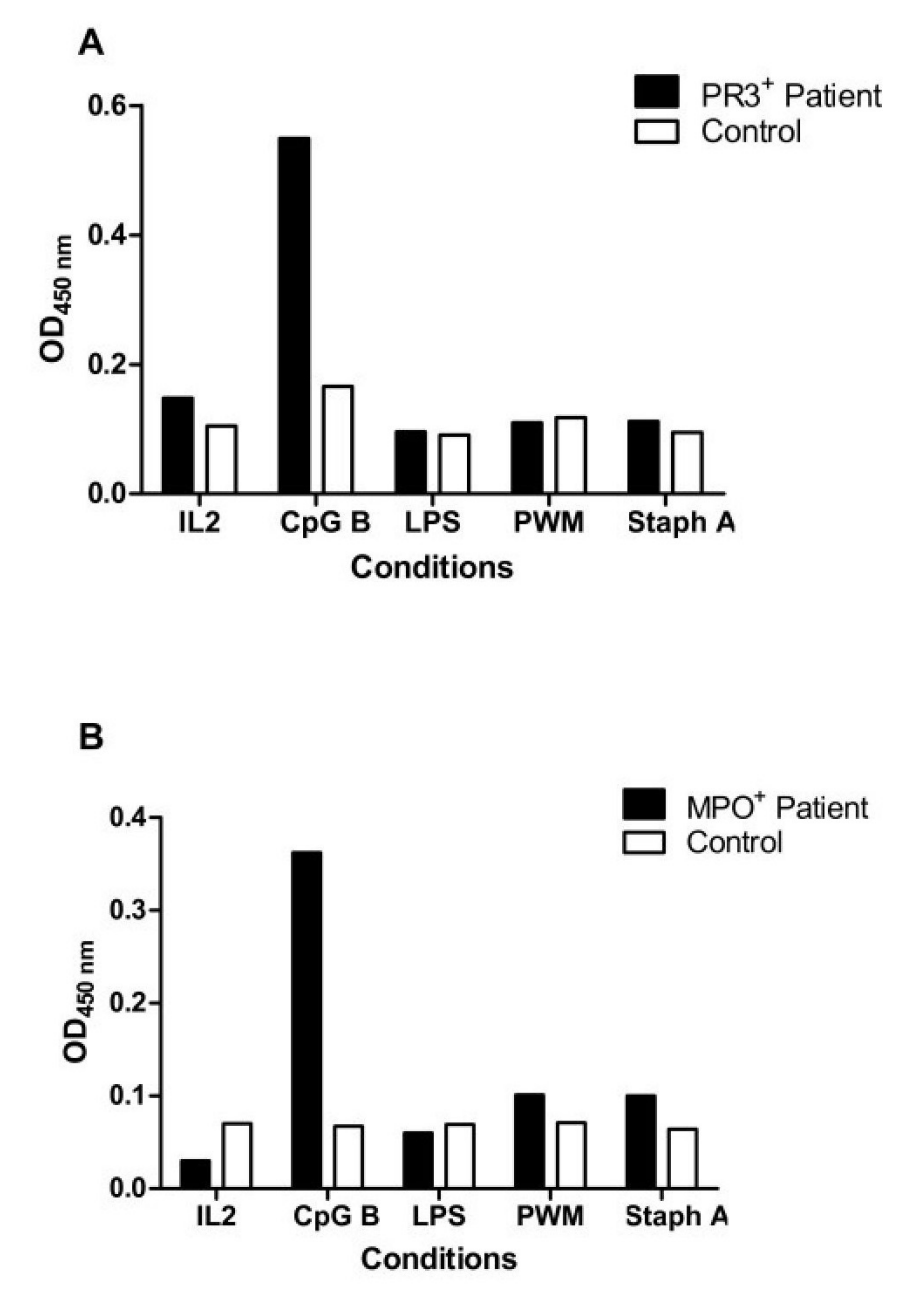
***In vitro* ANCA production is induced by CpG-B but not by other B cell stimulants**. In addition to CpG-B + IL2, PBMCs from 5 different pairs were also stimulated with either IL2 alone, LPS + IL2, pokeweed mitogen (PWM) + IL2, or inactivated *staphylococcus aureus *+ IL2. A representative set of results is shown from a PR3^+ ^ANCA patient (**A) **and a MPO^+ ^ANCA patient (**B**).

PBMCs were isolated from 10 patients with biopsy-proven ANCA+ vasculitis, each paired to a normal control individual, and cultured in the presence of CpG-B and IL-2. Patient clinical details are shown in Table 1. Notably, all but one of these patients were not receiving immunosuppressive medications at the time of assay. Cell culture supernatants were harvested after 12 days of culture, and concentration of IgG measured by ELISA as well as their reactivity against PR3 or MPO (Fig. 1). The supernatants from PR3+ and MPO+ ANCA patients were tested for reactivity against PR3 or MPO antigen respectively. We found that CpG B at 3.2 mg μg mL-1 to be a strong stimulant of IgG production *in vitro*. The amount of IgG detected in the supernatants were similar in both patient and control groups, with a concentration of 5.5 ± 2.2 and 4.1 ± 1.2 μg mL-1 respectively (Fig. 1A). The reactivity against PR3 and MPO antigens in the patients' supernatants were consistently greater than those observed in normal controls. In PR3+ ANCA patients, this difference achieved significant levels (P = 0.0082) (Fig. 1B). In MPO+ patients the difference was not significant (P = 0.072) although their supernatants showed a clear tendency towards higher reactivity (Fig. 1C). There was no correlation between serum ANCA titre and the amount of ANCA produced *in vitro *(r^2 ^= 0.172) (Fig. 1D). In addition to CpG-B, PBMCs from 5 different pairs were also stimulated with LPS, pokeweed mitogen (PWM) or inactivated *staphylococcus aureus *(Fig. 2). The production of IgG under these conditions was significantly lower compared to CpG-B and we were not able to detect ANCA in these conditions. The inactivated staphylococcus aureus used in these experiments had been formalin fixed. In this form, they are unlikely to provide freely available unmethylated CpG, even though they should contain unmethylated CpG within.

**Table 1:** Clinical and laboratory data of the patients at the time of study

**Patient Number**	**Age**	**Gender**	**ANCA Titre**	**Clinical Status**	**Treatment**	***Organ Involvement***
1	37	M	PR3 60 U	Remission	Nil	R, J
2	69	M	PR3 34 U	Newly Diagnosed	Prednisolone 5 mg/day	R, J
3	56	M	PR3 >100 U	Relapse	Nil	R, J, S, P
4	73	F	PR3 >100 U	Newly Diagnosed	Nil	R, L, E
5	50	M	PR3 33 U	Newly Diagnosed	Nil	L, E, J
6	63	M	MPO >100 U	Newly Diagnosed	Nil	R, L, C, J
7	77	M	MPO 54 U	Newly Diagnosed	Nil	R, L
8	67	M	MPO 38 U	Relapse	Nil	R, L
9	57	M	MPO 47U	Relapse	Nil	R, S, L
10	82	M	MPO >100 U	Newly Diagnosed	Nil	R, J

R = Renal, E = ENT, L = Lung, C = Cardiac, J = Joints, S = Skin, P = Peripheral Neuropathy

### Detection of peripheral blood circulating B cells capable of producing ANCA in response to CpG-B

**Figure 3:**
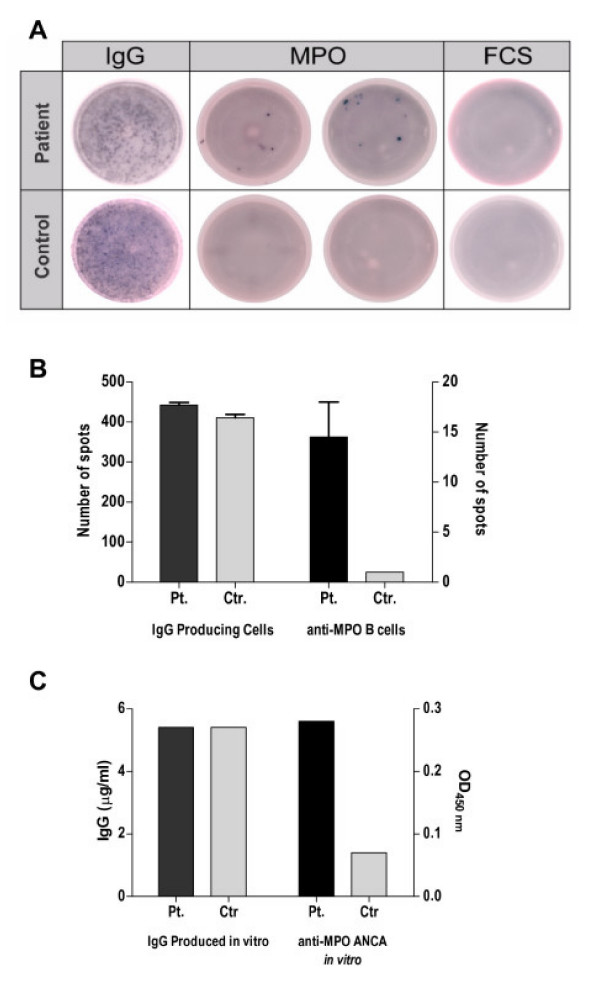
**Detection of circulating B cells capable of producing ANCA in response to CpG-B**. PBMCs from 2 MPO^+ ^ANCA vasculitis patients were cultured with CpG-B and IL-2. These PBMCs had not undergone enrichment for B cells prior to culture. After 5 days culture, cells were transferred into ELISpot wells which had been coated with either myeloperoxidase (MPO) in duplicates or foetal calf serum (FCS) as a control antigen. After overnight culture, IgG antibody producing cells against these antigens were detected by anti-human IgG conjugate. The total number of IgG producing B cells was measured by coating the wells with polyclonal anti-human IgG. The results from a patient as shown in this figure are representative of results from 2 patients. Fig **A **shows ELIspot plate with total IgG producing cells in the first column followed by the detection of anti-MPO B cells in duplicates in the middle columns and finally cells against the control antigen. The numbers of spots counted are depicted in Figure **B**. In spite of both patient and control having similar number of IgG producing cells, the number of anti-MPO B cells is higher in the MPO^+ ^patient. This result coincide with those from a parallel experiment where PBMCs from this pair of individuals were cultured in the presence of CpG-B to measure their *in vitro *production of anti-MPO by ELISA as shown in (**C**).

The production of ANCA autoantibodies by PBMCs in ANCA+ vasculitis patients suggested the presence of circulating ANCA autoreactive B cells in these patients. In order to test this possibility, we attempted to detect peripheral blood circulating B cells that are capable of producing ANCA by ELISpot. PBMCs isolated from a MPO+ ANCA patient who had had a relapse of vasculitis disease (patient no.8 in Table 1) were cultured with CpG-B and IL-2 for 5 days. Cells were then transferred into wells previously coated with either myeloperoxidase antigen or control antigen, and cultured overnight. Antibody producing cells that had produced IgG antibody against these antigens were detected by an anti-human IgG antibody. The ELISpot assay shows the presence of MPO-reactive B cells within the PBMC population of the MPO+ ANCA vasculitis patients but not of the control individuals (Fig. 3). Together, the above data indicate that ANCA+ vasculitis patients have in their peripheral circulation B cells which are capable of producing ANCA in response to CpG stimulation.

### CpG-B also induced production of the relevant IgG autoantibodies in patients with other autoimmune diseases *in vitro*

**Figure 4:**
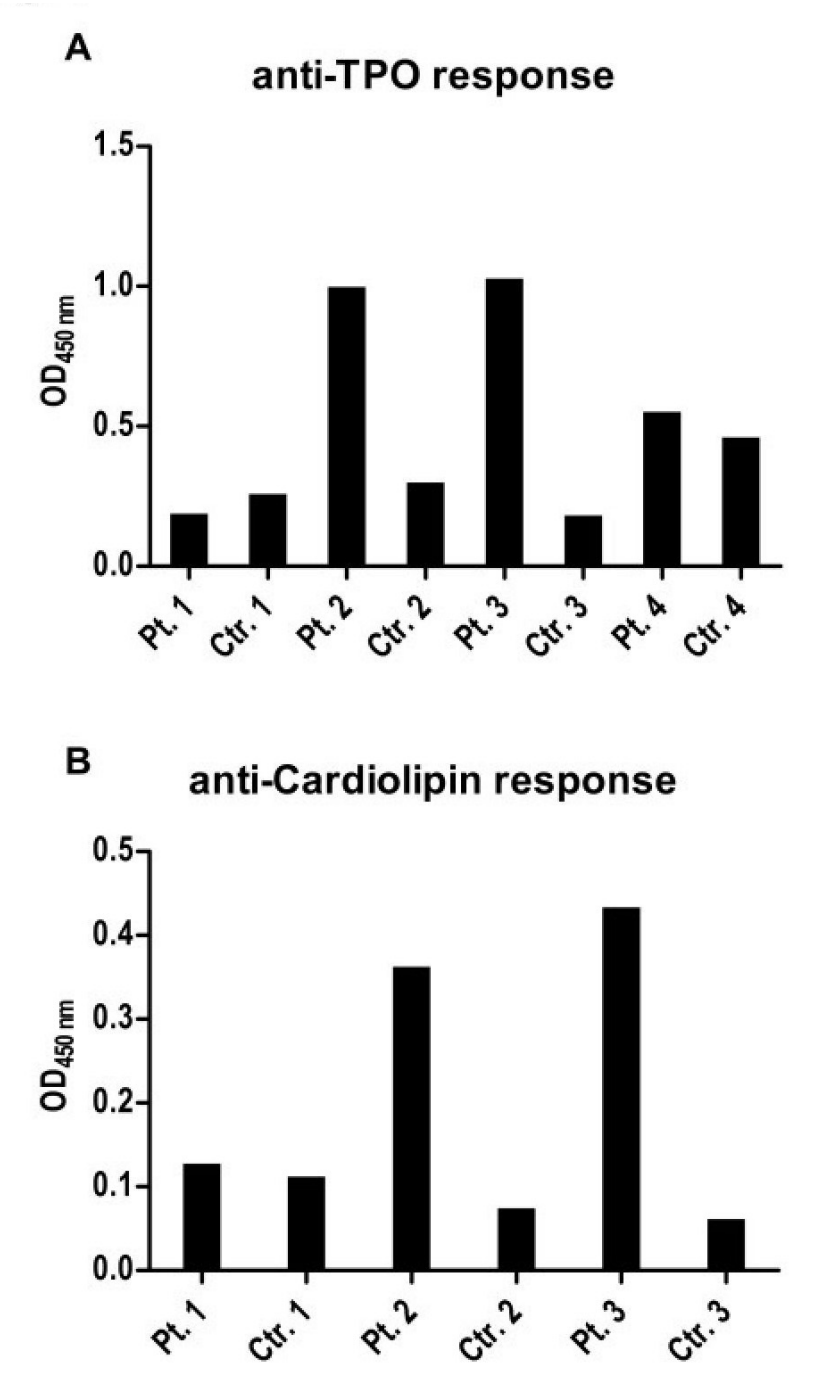
**CpG-B also induced the production of relevant IgG autoantibodies in patients with other autoimmune diseases**. PBMCs from 4 patients with autoimmune thyroiditis and 3 patients with anti-phospholipid antibody syndrome were cultured with CpG-B and IL-2. Each patient assay was paired with a healthy control. After 12 days of culture, supernatants from autoimmune thyroiditis patients were tested by ELISA for IgG anti-thyroperoxidase (TPO) as shown in **A**. Supernatants from anti-phospholipid antibody syndrome patients were tested for IgG anti-cardiolipin antibodies (ACLA) as shown in **B**.

To test if the CpG-B effect of inducing autoantibody production may be seen in other autoimmune diseases besides ANCA+ vasculitis, the same experimental procedure was performed in patients presenting with other types of autoimmune diseases, namely autoimmune thyroiditis and anti-phosphoslipid antibody syndrome. These patients were similar to most of our ANCA+ vasculitis patients in that they were not taking immunosuppressive medications at the time of study. We observed that stimulation of PBMCs with CpG-B and IL-2 led to the production of IgG anti-thyroperoxidase and anti-cardiolipin antibodies from the majority of patients with thyroiditis (3 out of 4 patients) and anti-phosphoslipid antibody syndrome respectively (2 out of 3 patients) (Fig. 4). These patients did not produce ANCA after CpG-B stimulation *in vitro *(data not shown).

## Discussion

It is known that low affinity autoreactive B cells are part of our normal immune repertoire [29], and several studies have demonstrated the presence of circulating autoreactive B cells in autoimmune diseases such as autoimmune thyroiditis [30] and systemic lupus erythematosus (SLE) [31]. In human SLE, Boumpas and colleagues have recently reported the finding of an increased number of TLR-9-expressing memory B cells and plasma cells in patients with active disease [32]. This is highly relevant to the report by Marshak-Rothstein and colleagues who had shown that co-engagement of BCR and TLR-9 by complexes of anti-DNA antibody and DNA may provide a mechanism for stimulation of autoreactive B cells in lupus [33]. This demonstration becomes even more relevant given that patients with SLE have impaired DNA methylation [34,35]. It may also provide a tangible explanation for the beneficial effect of agents like hydroxychloroquine that inhibit TLR-9 signaling by disturbing endosomal acidification.

Similar to thyroiditis and SLE, we have also detected circulating ANCA autoreactive B cells in patients with ANCA^+ ^vasculitis. In addition, we demonstrate that these cells are capable of producing ANCA autoantibodies in response to molecular ligands derived from bacteria and viruses such as CpG ODN. These findings provide experimental support for a link between infection and development of ANCA autoantibody.

Lanzavecchia and colleagues have reported that memory B cells proliferated and differentiated into antibody-secreting cells in response to CpG even in the absence of specific antigen stimulation [36]. They showed that CpG led to polyclonal activation of B cells against a range of vaccine and pathogen derived antigens, and they proposed that this microbial stimulus provided a mechanism whereby serological memory of human memory B cells is maintained. In the case of ANCA associated vasculitis, we propose a parallel mechanism, whereby microbial CpG ODN may contribute to the maintenance of ANCA autoreactive memory B cells and ANCA autoantibody production in patients with ANCA associated vasculitis.

Our findings that CpG-B stimulates the production of the relevant autoantibodies *in vitro *in patients with ANCA associated vasculitis, autoimmune thyroiditis and anti-phospholipid antibody syndrome suggest that this mechanism may also apply to the maintenance of autoreactive memory B cells in other antibody mediated autoimmune diseases.

Besides being the target of ANCA autoantibodies, neutrophils may also play an accessory role to drive the expansion of ANCA reactive B cells. Recently, Brinkmann and colleagues showed that activated human neutrophils release DNA to form neutrophil extracellular traps (NETs) [37,38]. The function of NETs is to ensnare and inactivate bacteria in defense against invading infection. Interestingly, the DNA in NETs is integrated with neutrophil antigens such as myeloperoxidase, elastase and cathepsin G. Given the highly cationic nature of these molecules (for example, the pI values of PR3, MPO, elastase, cathepsin G are 7.79, 9.22, 9.89 and 11.3 respectively), there is a theoretical possibility that neutrophil DNA from NETs or CpG sequences derived from bacteria caught in NETs may preferentially stimulate ANCA reactive B cells by virtue of electrostatic association between negatively-charged DNA and these cationic neutrophil antigens. This may then result in preferential expansion of ANCA reactive B cells. For instance, highly cationic MPO may bind non-covalently to negatively charged DNA containing hypomethylated CpG sequences. After encounter with anti-MPO specific autoreactive B cells, complexes of cationic MPO/CpG may become internalised, and CpG-B may subsequently stimulate TLR-9 within endosomes. This concept is supported by a recent demonstration that internalization of BCR antigen complex triggers the fusion of endocytic vesicles with TLR-9 containing vesicles [39].

Another potential endogenous source of stimulatory CpG could be latent viruses. Lund and colleagues have shown that viral DNA from Herpes simplex virus can stimulate murine TLR-9 [40]. Since the majority of individuals harbour herpes viruses and cytomegalovirus by adulthood, and circulating viral DNA is detectable in serum, it would be relevant to see if these viruses may provide a source of hypomethylated CpG ODN to maintain autoreactive B cells in patients with ANCA associated vasculitis.

Since the first description of circulating ANCA in patients with Wegener's Granulomatosis, the vast majority of research in this field has focused on the biological functions of ANCA and whether they have a pathogenic role in disease. In contrast, much less attention has been paid to the role of ANCA specific circulating B cells in this disease. Several studies have reported the successful use of Rituximab in patients with active disease [5,41]. The clinical response to Rituximab is generally rapid. Interestingly, the clinical improvement correlates with the depletion of circulating B cells, but not immediately with the serum ANCA titre, which may remain high for much longer before decreasing. This observation makes us suspect that ANCA-specific autoreactive B cells may possess additional pathogenic roles in this disease above and beyond the production of ANCA autoantibodies, such as antigen presentation and cytokine production. Hence, it would be equally worthwhile to investigate if exposure of B cells to pathogen-derived CpG may stimulate these functions in vasculitis.

## Conclusion

Circulating ANCA autoreactive B cells are present in patients with ANCA^+ ^vasculitis. These cells are able to produce measurable amounts of ANCA *in vitro*. This response seems to be limited to stimulation with CpG oligodeoxynucleotide, as we could not detect ANCA in response to other B cell stimulants such as LPS, pokeweed mitogen or *staphylococcus aureus*. These findings lead us to propose that stimulation of ANCA autoreactive B cells by immunostimulatory DNA sequences such as CpG during infection may provide a link between infection and ANCA associated vasculitis. This phenomenon may also operate in other antibody mediated autoimmune diseases. Further identification of the nature of immunostimulatory oligodeoxynucleotides and ANCA autoreactive B cells in patients may offer a greater understanding of the mechanisms by which infection is associated with development of this disease. The question of whether infection may stimulate *de novo *production of ANCA in individuals without vasculitis also warrants separate investigation. This possibility will need to be addressed by examining a large number of patients with different infections as the propensity to produce ANCA in response to infection may depend not only on the type of infection in question, but also on the genetic predisposition of the infected individual.

## Methods

### PBMC separation and culture

25 mls of peripheral blood was obtained with consent from ANCA+ vasculitis patients with biopsy proven disease. Consent was obtained in accordance to the ethical standards specified by the Royal Adelaide Hospital Human Ethics Committee. PBMC separation was performed by density gradient over LymphoprepTM (Axis-Shield, Oslo). After extensive washing, cells were resuspended in culture media (RPMI 1640 supplemented with 10% foetal calf serum, 2 mM L-glutamine, 100 U mL^-1 ^penicillin, 100 mg mL^-1 ^gentamicin) and adjusted to a final concentration of 1 × 10^6 ^cells mL^-1^. Each patient sample was prepared in parallel with a control sample obtained at the same time from a healthy individual. PBMCs were stimulated with 3.2 μg mL^-1 ^of CpG-B ODN 2006 (5'-t**cg**t**cg**ttttgt**cg**ttttgt**cg**tt-3'), 3.2 ug mL^-1 ^CpG-A ODN 2216 (5'-ggGGGA**CG**AT**CG**TCgggggG-3'; small letters, phosphorothioate linkage; bold, CpG dinucleotides; capital letters, phosphodiester linkage 3' of the base; Geneworks, Australia), IL-2 10 ng mL^-1 ^(R&D Systems), LPS 500 ng/ml (Sigma-Aldrich), pokeweed mitogen 3 μg mL^-1 ^(Sigma-Aldrich), inactivated *staphylococcus aureus *Cowan's strain (Sigma-Aldrich) or media alone. All solutions had been tested free of endotoxin. CpG was free of endotoxin (< 0.1 EU/ml) while inactivated staphylococcus aureus contained 1.53 EU/ml (LAL method, Charles River PTS System). Cell culture supernatant was harvested after 12 days of culture at 37°C, 5% CO2.

### ELISA

Total IgG concentrations in cell culture supernatants were measured by ELISA (Bethyl Laboratories) while ANCA specificity was measured using ELISA to PR3, MPO, thyroperoxidase or cardiolipin (Orgentec, Germany). To detect IgG ANCA in supernatant, samples were diluted 1:2 and incubated for 1 hr, followed by 1 hr with anti-human IgG conjugated to horseradish peroxidase. Enzyme activity was detected using TMB substrate, stopped with hydrochloric acid and read at 450 nm.

### ELISpot

PBMCs were cultured at 2 × 106 cells mL^-1 ^in RPMI 1640 supplemented with 10% foetal calf serum, 2 mM L-glutamine, 100 U mL^-1 ^penicillin, 100 mg mL^-1 ^gentamicin, in the presence of CpG B 3.2 μg mL^-1 ^and IL-2, at 37°C in 5% CO2 for 5 days. Twenty-four hours prior to harvesting the cells, MultiScreen HTS 96 wells plate (Millipore, France) was activated with 15 μl of 70% methanol for two minutes and washed once with endotoxin-free PBS. The wells were coated with 100 μl of either goat anti-human IgG (Jackson ImmunoResearch), 20 μg mL^-1 ^of purified myeloperoxidase (Calbiochem, Germany) or 10% foetal calf serum, all diluted in carbonate/bicarbonate buffer 0.15 M, pH 9.6. The plates were washed and blocked with 100 μl of complete media and incubated for 90 min at 37°C. Meanwhile, cells were collected, washed 4× in complete media and adjusted to a final cell density of 2.5 × 10^6 ^μL mL^-1^. 100 μL well^-1 ^of the cell suspension was placed in the ELISpot plate in duplicate and cultured for further 12 hrs. Plates were washed with PBS and incubated with 100 μl of goat anti-human IgG conjugated to alkaline phosphatase (Sigma, Germany) diluted 1:3000, at 4°C overnight. Plates were washed and developed by adding 100 μL well^-1 ^of BCIP/NBT Plus (Mabtech, Sweeden). The reaction was stopped at 8 min by rinsing the plate with running water. Plates were read by AID plate reader (Autoimmune Diagnostica, Germany).

### Statistics analysis

Data are presented as means ± standard error of the mean (SEM), and the significant differences were determined using Student's *t *test using GraphPad Prism Software [42].

## Authors' contributions

PRH, LJ, DG and CAP conceived the study, performed the experiments and wrote the manuscript. JN, MP, GS, JC and PH assisted in patient sample collection and processing.
